# Decrypting the Sequence of Structural Events during the Gating Transition of Pentameric Ligand-Gated Ion Channels Based on an Interpolated Elastic Network Model

**DOI:** 10.1371/journal.pcbi.1001046

**Published:** 2011-01-06

**Authors:** Wenjun Zheng, Anthony Auerbach

**Affiliations:** 1 Department of Physics, University at Buffalo, Buffalo, New York, United States of America; 2 Department of Physiology and Biophysics, University at Buffalo, Buffalo, New York, United States of America; New York University, United States of America

## Abstract

Despite many experimental and computational studies of the gating transition of pentameric ligand-gated ion channels (pLGICs), the structural basis of how ligand binding couples to channel gating remains unknown. By using a newly developed interpolated elastic network model (iENM), we have attempted to compute a likely transition pathway from the closed- to the open-channel conformation of pLGICs as captured by the crystal structures of two prokaryotic pLGICs. The iENM pathway predicts a sequence of structural events that begins at the ligand-binding loops and is followed by the displacements of two key loops (loop 2 and loop 7) at the interface between the extracellular and transmembrane domain, the tilting/bending of the pore-lining M2 helix, and subsequent movements of M4, M3 and M1 helices in the transmembrane domain. The predicted order of structural events is in broad agreement with the Φ-value analysis of α subunit of nicotinic acetylcholine receptor mutants, which supports a conserved core mechanism for ligand-gated channel opening in pLGICs. Further perturbation analysis has supported the critical role of certain intra-subunit and inter-subunit interactions in dictating the above sequence of events.

## Author Summary

Pentameric ligand-gated ion channels are a family of membrane proteins that open/close an ion-conducting channel in response to the binding of specific ligands. Some members of the family, including nicotinic acetylcholine receptors, play key physiological roles in signal transduction at synapses. Despite many experimental and computational studies of the gating transition of these pentameric ion channels, the structural basis of how ligand binding couples to channel opening remains uncertain. In particular, the all-atom computer simulation of the gating transition is limited to nanosecond ∼ microsecond time scales while the entire transition takes tens of microseconds. In this study, we have employed a highly efficient coarse-grained modeling method to dissect the sequence of structural events underlying the gating transition. The model predictions are in broad agreement with the kinetic analysis of mutants of nicotinic acetylcholine receptors. This study has established a useful computational framework to simulate the functional dynamics of pentameric ligand-gated ion channels.

## Introduction

Pentameric ligand-gated ion channels (pLGICs) are a family of membrane proteins that open/close an ion-conducting channel in response to an increase/decrease in the binding affinity for specific ligands [1], [2], [3], [4]. Some members of the family, including nicotinic acetylcholine receptors (AChRs, [5]), play key physiological roles in signal transduction at synapses.

The pLGICs share the common structural architecture of a pentamer with each subunit consisting of an extracellular ligand-binding domain (ECD) and a transmembrane channel domain (TMD). The ligand-binding sites lie at the interfaces between adjacent ECDs and the TMD of each subunit is comprised of four transmembrane helices, M1–M4. Recent structural investigations have yielded several atomic or near-atomic structural models of pLGICs, including a 4 Å-resolution refined model of the *Torpedo* AChR obtained by electron microscopy [6], [7], the crystal structures of acetylcholine-binding proteins (AChBP) [8], [9], [10], the ECD of mouse AChR α subunit [11], and bacterial pLGICs from *Erwinia chrysanthemi* (ELIC) and the cyanobacterium *Gloebacter violaceus* (GLIC) [12], [13], [14], [15]. The crystal structures of ELIC [12] and GLIC [13], [14] may represent the low-affinity, closed-channel and high-affinity, open-channel conformations of the pLGICs, respectively. Despite their moderate sequence similarity (<20% sequence identity), the two proteins are highly similar in both secondary and tertiary structures [13], [14]. A comparison of the ELIC and GLIC structures offers the possibility of a detailed view to the global and local structural changes associated with the gating transition of pLGICs despite their variation in bound ligand (ELIC is gated by an unknown ligand, and GLIC is gated by proton instead of a neurotransmitter).

Various mechanistic models for the gating transition of pLGICs have emerged from a wealth of experimental data and structure-based simulations. It has been suggested that agonist binding initiates various conformational changes, including the movements of binding site loops A and B [16], loop C [17], [18], [19], [20] and loop F [20], a quaternary twist motion [21] and a tertiary deformation within the ECD [7]. These structural changes are thought to propagate to the TMD and cause either rotation [21], [22] or tilting [13], [14], [23], [24] of the pore-lining M2 helix, which leads to the opening of the physical gate formed by the bulky side chains of hydrophobic residues in the equatorial region of the M2 helix [12]. Although multiple interface loops, secondary structure elements, and key residues have been implicated in the signal transmission from ECD to TMD (see [19], [25], [26], [27], [28]), the full details of the signaling pathway are not known with certainty. To explore the molecular mechanism of signaling in AChRs, single-channel kinetic and rate-equilibrium free energy relationships (Φ-value analysis) of mutant AChRs have been analyzed [3], which has led to the proposal that the gating occurs as a conformational cascade that propagates from the ligand-binding site to the channel pore via sequential, coupled movements of rigid-body blocks with distinct Φ-values [29]. The nature of these structural motions is thought to be stochastic Brownian motions [30] although the details remain to be worked out. It is likely that one or more of the intermediate states of this conformational pathway has been detected in high-resolution patch-clamp experiments [31].

The gating transition of pLGICs has been studied extensively by a variety of computational methods, including equilibrium molecular dynamics (MD) simulation [17], [18], [32], [33], [34], [35], [36], [37], targeted MD simulation [27], Brownian dynamics simulation [38], and normal mode analysis (NMA) [21], [39], [40], [41]. Nevertheless, atomistic MD simulations of protein dynamics are limited to a time range of nanoseconds ∼ microseconds [42] despite fast advancing computing technology. Although MD simulations ranging from tens of nanoseconds (see [27], [32]) to one microsecond [37] have revealed interesting conformational changes that may lead to channel opening/closing, the simulation times remain far less than the 10∼20 µs time range necessary for the activation of neuromuscular AChRs [43].

To overcome the time-scale barrier for MD simulations, a variety of coarse-grained models [44] have been developed to simulate protein conformational dynamics with greater efficiency. Of particular interest to the present study is the elastic network model (ENM) [45], [46], [47], which represents a protein structure as a network of C_α_ atoms with neighboring ones connected by springs with a uniform force constant [48]. The normal mode analysis (NMA) of ENM often yields a handful of low-frequency modes that dominate the large-scale conformational changes observed between two protein crystal structures [47], [49]. Numerous studies have established ENM as an efficient and robust means to tease out the functionally relevant conformational dynamics from protein structures with no limit in time scale or system size (for reviews, see [50], [51], [52]). Indeed, ENM has formed the basis of several computational methods for modeling protein conformational transitions [53], [54], [55], [56]. In an earlier study [55], one of us developed the mixed-ENM technique to generate a transition pathway between two given conformations using a double-well mixed-ENM potential, which is built from two ENM potentials constructed based on the two given conformations. A similar approach (plastic network model) was proposed by Maragakis and Karplus [54]. In another related study by Delarue and coworkers, a transition pathway was generated by minimizing an ENM-based action function [56]. Recently, Zhu and Hummer applied the mixed-ENM method to the gating transition of the TMD of pLGICs [57]. They found that the conformational transition involves a concerted tilting of helices M2 and M3, and M2 changes its bending state, which results in an early closure of the channel pore during the open-to-close transition [57]. Despite the above insights, the conformational transition of the full pLGIC (including both ECD and TMD) remains to be simulated to determine the sequence of structural events that couple ligand binding to channel gating.

Recently, one of us has developed an interpolated-ENM (iENM) method based on the mixed-ENM method to predict a likely transition pathway from the beginning conformation to the end conformation of a transition [58]. Compared with the mixed-ENM method, which is based on an approximate solution of saddle points of the mixed-ENM potential [55], the iENM method solves the saddle points exactly and efficiently by iteratively calling a sparse linear-equation solver [58]. Such improvement has led to better prediction of the order of local and global structural changes as validated by experimental structural data [58]. We have used iENM to compute a possible transition pathway from the closed-channel conformation to the open-channel conformation of pLGICs as captured by the crystal structures of ELIC and GLIC, respectively. The iENM pathway predicts a sequence of structural events beginning with the movements of ligand-binding loops, and is followed by the displacements of loop 2 and loop 7 at the TMD-ECD interface, the tilting/bending of pore-lining M2 helix, and subsequent movements of M4, M3 and M1 helices. The predicted order of structural events is in general agreement with the Φ-value analysis of AChR mutants, which supports a conserved core mechanism for ligand-gated channel opening in pLGICs. Further perturbation analysis has supported the critical role of certain intra-subunit and inter-subunit interactions in dictating the above sequence of events.

## Results

We will first discuss the results of ENM-based NMA on the ELIC structure, which will motivate the modeling of the gating transition of pLGICs beyond single-mode description. Next, we will perform the iENM-based transition pathway modeling of the gating transition, and compare the results with the Φ-value analysis. Finally, we will employ perturbation analysis to identify the key interactions that dictate the specific order of structural events predicted by iENM.

### NMA of ELIC structure

Previous NMA studies have found that the lowest normal mode captures a quaternary twist motion of the homo-pentameric α7 nAChR with opposing rotations of the ECD and TMD, which is accompanied by reorganizations within subunits and opening of the channel pore [21], [40]. To explore if similar conformational changes are favored by the ELIC crystal structure, we have performed ENM-based NMA (the cutoff distance *R_c_* is chosen to be 10 Å, which maximizes the cumulative overlap between the lowest 1% normal modes and the observed conformational change from ELIC to GLIC structures).

Indeed, the observed conformational change overlaps significantly with the first normal mode (overlap = 0.54), which describes a quaternary twist motion of ECD relative to TMD [14]. To evaluate if this mode facilitates channel opening, we have generated a new ELIC conformation after displacing the C_α_ atoms along the direction of the eigenvector of this mode by an RMSD of 3 Å (note: the RMSD between ELIC and GLIC structures is ∼3.1 Å). Then we use the HOLE program [59] to calculate the radius profiles of the channel pore formed by the C_α_ atoms only (each C_α_ atom is assigned an atomic radius of 3 Å following [57]). The minimal pore radius is found to be nearly unchanged after the displacement along the first mode (∼0.007 Å), which indicates no opening of the ion-conducting channel. Therefore, unlike α7 nAChR, the first normal mode alone does not support a coupling between the quaternary twist motion and the opening of the channel pore in ELIC. The same observation was made in another NMA study of ELIC structure based on an all-atom force field [36]. Therefore, the single-mode description of ELIC dynamics does not fully support the “twist-to-open” model of the gating transition of pLGICs [21]. Indeed, much of the observed conformational change from ELIC to GLIC structures is not captured by one or a few lowest modes (only 44% is captured by the lowest 1% or 45 normal modes) (see [14]). Therefore, it is necessary to incorporate more normal modes to accurately model the conformational transition that leads to channel opening in pLGICs.

### Transition pathway modeling by iENM

**Figure 1:**
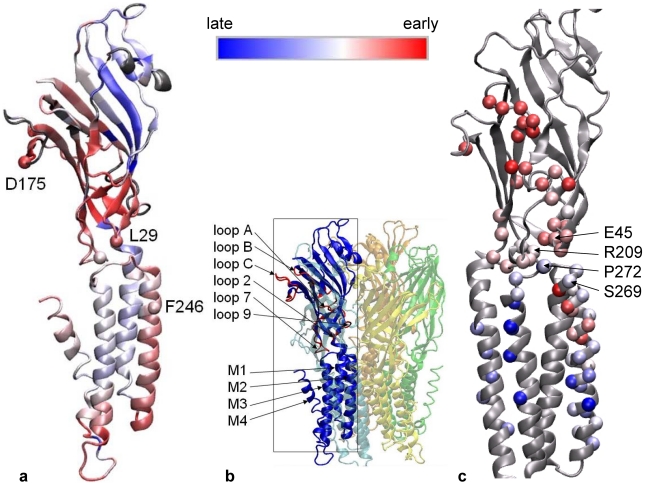
Results of iENM modeling of the gating transition between the closed-channel ELIC structure and the open-channel GLIC structure in comparison with experimental Φ values of the nAChR α subunit. (**a**). Residues of an ELIC subunit colored by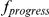 (early/intermediate/late-moving residues are colored red/white/blue). (**b**). The structural architecture of ELIC pentamer with key structural elements labeled and key loops (loops A, B, C, 2, 7, 9) colored in red. (**c**). A subset of residues of the nAChR α subunit shown as spheres and colored by 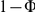 (early/intermediate/late-moving residues are colored red/white/blue).

The iENM method [58] enables the simulation of a conformational transition between two given conformations by implicitly utilizing all normal modes from NMA. The iENM method generates a possible transition pathway by solving a set of saddle points for an interpolated potential function constructed from the two ENM potentials based at the beginning and end conformations of a transition (see Methods). We have applied iENM to the conformational transition from the ELIC structure to the GLIC structure to simulate the ligand-gated transition of pLGICs. The resulting iENM pathway consists of 54 intermediate conformations (sampled at an RMSD increment ≥0.1 Å, for a movie see Video S1 of Supporting Information). To dissect the motional order of individual residues, we have calculated a 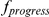 parameter (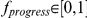) for each residue (low/high 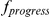
means early/late movement, see Methods). The residues of ELIC are colored according to 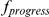
(residues with low/medium/high 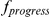
are colored red/white/blue, see Fig. 1a). The distribution of 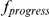
supports the following motional order of structural elements: loops A and C
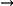
loop 2
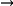
loop 7, M2 helix
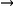
M4 helix
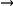
M3 helix
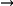
M1 helix (listed in the order of increasing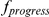, see Table 1). Therefore, we can deduce a sequence of structural events beginning with the closing of ligand-binding loops in ECD (including loops A and C), followed by the displacements of loop 2 and loop 7 at the TMD-ECD interface, then the tilting/bending of M2 helix, and later the movements of M4, M3 and M1 helices.

**Table 1:** The average 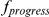 for key structural elements of ELIC calculated by iENM without/with perturbation to selected interactions.

		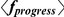 with perturbation to					
key elements	residue range	none	TMD-ECD	inter-subunit	loop 2	loop 7	loop 9
loop A	79–82	0.176	0.329	0.072	0.216	0.177	0.177
loop B	132–137	0.360	0.400	0.373	0.366	0.362	0.362
loop C	174–185	0.208	0.204	0.097	0.200	0.210	0.209
loop 2	28–31	0.225	0.264	0.180	0.256	0.227	0.226
loop 7	112–122	0.294	0.326	0.320	0.346	0.355	0.295
loop 9	147–159	0.264	0.198	0.222	0.245	0.267	0.266
M1	201–219	0.495	0.557	0.372	0.484	0.490	0.495
M2	227–251	0.302	0.430	0.461	0.349	0.309	0.304
M3	260–282	0.436	0.548	0.430	0.435	0.437	0.438
M4	296–316	0.390	0.554	0.303	0.393	0.366	0.392
M2–M3 linker	252–259	0.355	0.359	0.648	0.354	0.387	0.354

To validate the iENM modeling results, we have compared our prediction of sequence of structural events with the Φ-value analysis of the α subunit of AChR mutants [3]. Despite the tremendous differences between the two approaches (the former is based on coarse-grained structural simulation, while the latter is based on kinetic analysis of mutated proteins), we have found broad agreement between their predicted order of structural events during the close-to-open transition of pLGICs summarized as follows (note: unlike 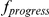, low/high Φ value implies late/early motion):

1. Early movements of ligand-binding loops A, B and C (Φ∼0.93, [16]) followed by loops 2 and 7 (Φ∼0.75, [29], [60]);
2. A sequence of motions in transmembrane helices in the order M2 (Φ ∼0.65, [29], [30]) 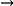
M4 (Φ ∼0.54, [61]) 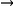
M3 (Φ∼0.32, [62]);
3. A sharp transition from an early-moving residue (R209, Φ∼0.74) to a late-moving residue (L210, Φ∼0.35) in the pre-M1 region ([63]);
4. A gradual transition from early-moving residues to late-moving residues along the M2–M3 linker, which agrees with the finding that the gating motions at the top of M2 helix occur before those at the top of M3 helix ([64]) based on, in part, the following mutational studies of AChRs ― mutation of αS269 increases K_eq_ mainly by increasing the channel opening rate constant ([30], [65]), and mutation of αY277 increases K_eq_ mainly by decreasing the channel closing rate constant ([62]).

**Figure 2:**
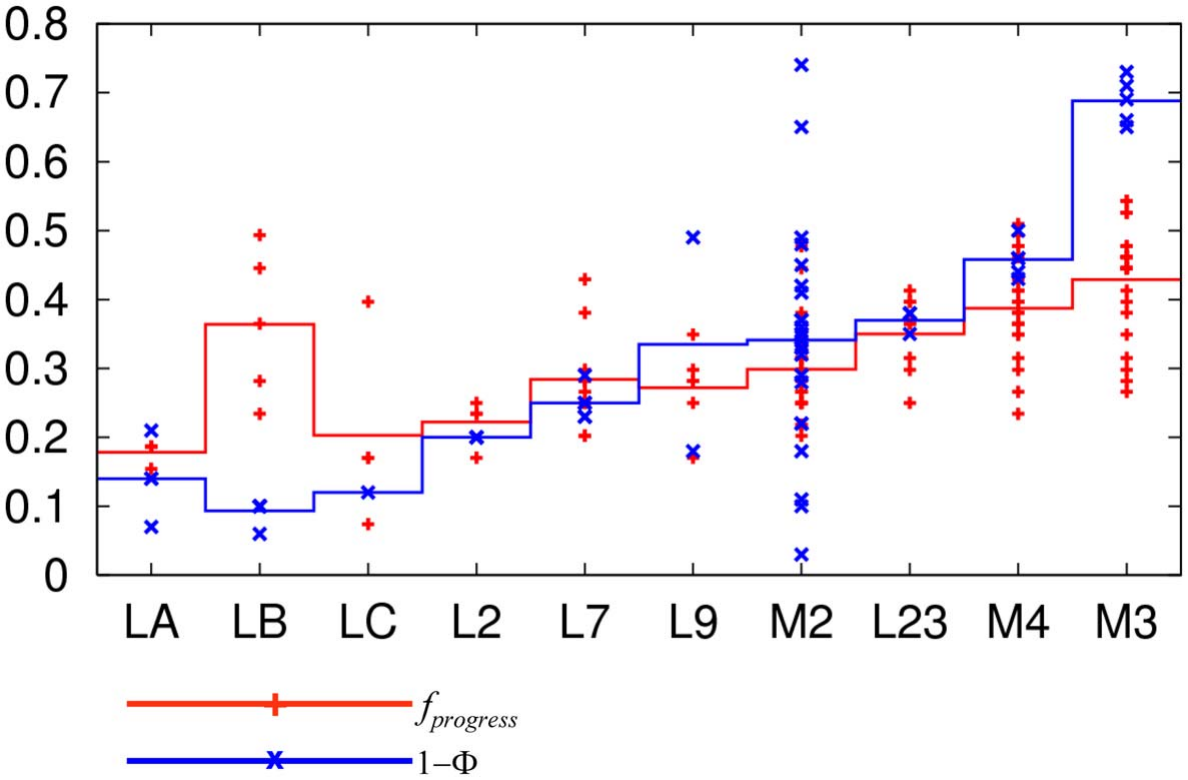
Comparison of 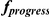
and 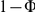
averaged over residues of 10 secondary structure motifs (loops A, B, C, 2, 7, 9, and helices M2, M3, M4, and M2–M3 linker). The average 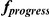
and 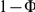 are shown as plateaus colored in red and blue, respectively. The values of 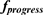
and 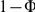
for individual residues within each motif are shown as + and x, respectively.

To further quantify the comparison between the theoretical 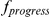
and experimental Φ values, we have averaged 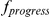 and 1-Φ over residues of 10 secondary structure motifs (loops A, B, C, 2, 7, 9, and helices M2, M3, M4, and M2–M3 linker, see Fig. 2). We do not include residues of M1 helix for the lack of experimental Φ values. The cross-correlation coefficient between the average 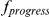 and 1-Φ is 0.73. As seen from Fig. 2, both of them follow a series of “ascending staircases” from loop A to M3 helix, with the only significant disagreement at loop B (if we remove loop B, the cross-correlation coefficient jumps to 0.95). Possible reasons for this disagreement are: 1. the ligand-binding loops (including loop B) are not well conserved between AChRs and ELIC, so their dynamics may differ; 2. there is a gap in the structural alignment of loop B between ELIC and GLIC (at residue 132, see Fig. S1 of Supporting Information), which may cause inaccuracy in modeling. Further studies are needed to resolve the above possibilities. Additionally, the variations of 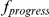 and Φ values do not seem to agree (see Fig. 2), especially in M2 helix where large scatter in Φ values were found [66]. This disagreement may be attributed to either the model limitation (such as the lack of sidechain and solvation) or the functional divergence between ELIC and AChRs.

### Perturbation analysis of transition pathway

To explore the intra-subunit and inter-subunit interactions that may dictate the sequence of structural events predicted by iENM, we have combined iENM with a perturbation analysis ― namely, we perform iENM after turning off the elastic interactions between selected sets of residues, and then analyze how the 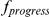 values of key structural elements change in response to such perturbation. The results are summarized as follows (see Table 1).

First, after turning off the intra-subunit interactions between TMD and ECD, the 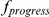 values of all transmembrane helices (M1–M4) increase significantly, which support the importance of the TMD-ECD interactions in facilitating the motions of transmembrane helices following ligand binding. To further identify the key TMD-ECD interactions, we have turned off those TMD-ECD interactions which involve loop 2, loop 7 and loop 9, respectively. We have found the 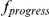 values of M2 helix increase significantly following the perturbation to loop 2 (but not loop 7 or 9, see Table 1). This finding supports the primary role of loop 2 in coupling ECD with the pore-lining M2 helix, while loop 7 and loop 9 may play some auxiliary role.

Second, after turning off the inter-subunit interactions between the ECDs of adjacent subunits, the 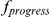 values of M2 helix increase significantly, while that of M1 and M4 helices decrease significantly (see Table 1). As a result, the motional order M2
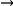
M4
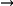
M3
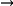
M1 is changed to M4
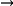
M1
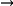
M3
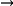
M2. Therefore, the inter-subunit interactions of ECDs are critical in controlling the sequential motions of transmembrane helices, which may allow ligand binding at the inter-subunit interfaces of ECD to activate or inhibit channel opening.

## Discussion

### Intermediate conformation of channel pore

**Figure 3:**
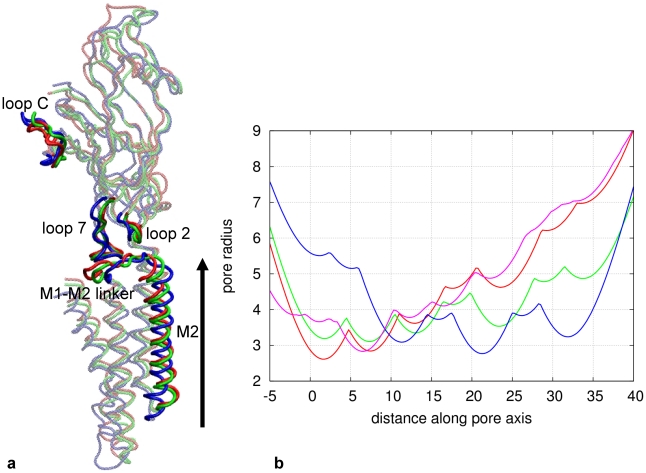
Comparison of the ELIC and GLIC structures with the intermediate conformation of iENM pathway. (**a**). Superposition of ELIC structure (blue), GLIC structure (red) and the intermediate conformation (green) at the middle of the iENM pathway (with 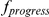 = 0.5), where key structural elements (loop C, loop 2, loop 7, M2–M3 linker and M2 helix) are opaque while the rest is transparent. (**b**). Pore radius as a function of distance along the pore axis (both in Å) for ELIC structure (blue), GLIC structure (red), nAChR structure (purple, [7]), and the intermediate conformation (green) at the middle of the iENM pathway (with 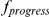 = 0.5). The pore axis is shown by an arrow in panel a.

As revealed by structural comparison, the M2 and M3 helices in GLIC have tilted relative to ELIC as a rigid unit by about 9°. This rotation results in an outward movement of the helix pair away from the pore axis on the extracellular side and an inward movement towards the pore axis on the intracellular side of the channel (see Fig. 3a). Because the channel pore is lined by M2 helices, the pore constriction is shifted from the extracellular side to the intracellular side of the channel (see Fig. 3b). We have compared the intermediate pore conformations predicted by iENM and mixed-ENM method [57]. The mixed-ENM modeling of the TMD found that both the intracellular side and the extracellular side of the pore are closed at the middle of the transition pathway (with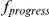 = 0.5), and both M2 and M3 helices undergo concerted tilting during the transition [57]. However, the iENM modeling of the entire pLGICs has found that the intracellular side of the pore is closed while the extracellular side of the pore is half-open at the middle of the transition pathway (see Fig. 3b), and the M2 helix moves earlier than the other transmembrane helices (M1, M3 and M4) (see Fig. 1a). Therefore, the modeling of both ECD and TMD is needed to elucidate how ligand binding facilitates the outward tilting of M2 helix followed by the motions of other transmembrane helices.

Our finding that M2 helix moves earlier than the rest of TMD implies a key coupling between M2 helix and ECD that forms early during the gating transition. This result agrees with previous proposals that the inner β-sheet of ECD is significantly correlated to the movement of M2 helix [21], and M2 helix moves independently from the other transmembrane helices [67].

### Primary ECD-TMD coupling via loop 2

Previous studies have suggested that several conserved loops (including loop 2, loop 7, loop 9, pre-M1 region, and M2–M3 linker, see [68]) at the ECD-TMD interface are involved in the signal transmission from ECD to TMD. It was proposed that loop 2 functions as an actuator that acts on the M2–M3 linker, while loop 7 may serve as a stator to bracket the rotation of M2 and M3 helices [18], [40]. Alternatively, both loop 2 and loop 7 may act together to coordinate the communication between ECD and TMD [69]. Based on the Φ-value analysis of AChR mutants, others suggested that a combination of side-chain interactions at several positions between loop 2 and M2 helix, and loop 7 and M2–M3 linker (specifically, P272 in the AChR) allows energy to be transferred from ECD to TMD [19], [25], [29].

Our finding supports the importance of the early-formed coupling between loop 2 and M2 helix, which is followed by structural rearrangements of loop 7 and M2–M3 linker. This result agrees with a recent MD simulation of ELIC [36], which found that the correlation between residues from loop 7 and M2–M3 linker is most prominent, while the correlation between loop 2 and loop 7 or M2–M3 linker is much weaker. The importance of loop 2 was also suggested by a Targeted MD simulation [27], which found that the closing of loop C transmits to the lower part of the *β*10 strand, which subsequently displaces loop 2 via the interaction between R209 and E45 (see Fig. 1c), and eventually drives the opening of channel pore [19], [40]. Our finding, rather than pinpointing a signaling path from the ligand-binding site to loop 2 via a chain of interactions [70], supports the collective involvement of a cluster of low-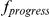 residues in the inner β-sheet (see Fig. 1a), which agree with the proposals that emphasized the collective motion of inner β-sheet [7], [13] and the involvement of a network of interactions including salt bridges [33] and electrostatic interactions [26] in controlling the gating process.

The coupling between loop 2 and M2 helix involves a conserved residue P253 (corresponding to P272 of *Torpedo* AChR or P269 of α7 AChR, see Fig. 1c). The conformational transition from ELIC to GLIC involves an inward displacement of the tip of loop 2 toward the pore center accompanied by an outward motion of the C-terminus of M2 helix (see Fig. 3a). In a targeted MD simulation of α7 AChR [27], the motion of loop 2 was sterically obstructed by M2–M3 linker (including P269). So the removal of the steric obstruction between these residues permits a rotation of the M2–M3 linker during the gating transition [27]. Similarly, Ref [6] proposed a ‘pin-into-socket’ model via a contact between loop 2 and the hydrophobic pocket formed by the end residues of the apposing M2 helix (αS269–α272 of Torpedo AChR, see Fig. 1c).

Although our modeling favors loop 2 over loop 7 as the primary element in coupling ECD with TMD, we cannot rule out possible loop-7-mediated coupling specific to eukaryotic pLGICs. Functional divergence of loop 7 is conceivable because this loop differs substantially between ELIC and nAChRs in both structure and sequence (see [36]).

### Structural interpretation of Φ-value analysis

The underlying structural picture of iENM modeling differs from the conformational cascade scenario proposed earlier [3]. The former involves a continuous energy-based interpolation between the ELIC and GLIC structures which features highly collective motions of various protein parts at different pace (as characterized by the 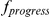
parameter), and the latter postulates Brownian motions of various protein parts in a discrete and stochastic fashion. The map of reaction progress obtained by using iENM modeling shares some general characteristics with the experimental map of Φ values in AChRs, but it does not show large spread in Φ values that have been revealed by kinetic analysis (see Fig. 2). The iENM and Brownian cascade models of gating represent extreme representations of the transition state ensemble. The former posits a single, frictionless barrier devoid of intermediate states while the latter holds that the barrier has rugged energy landscape that is populated by multiple, metastable intermediates. Future studies are needed to resolve the applicability of these two alternative mechanisms.

### Concluding remarks

High-resolution protein structures are critical for meaningful simulations of protein dynamics. Until recently, for the lack of high-resolution structures of full pLGICs, many MD and NMA simulations were conducted using homology models of pLGICs (see [18], [40]) with uncertain accuracy (see [33]). A main advantage of coarse-grained methods like iENM is that they are insensitive to atomic details and inaccuracy of initial structures. Additionally, the transition pathways predicted by iENM are independent of the specific form of the double-well potential function [58], and the biological relevance of the iENM-predicted pathways has been validated recently by structural data [58]. Therefore, iENM offers highly robust and efficient predictions for the dynamics of protein conformational transitions, including the gating transition of pLGICs. Compared with previous NMA studies based on a single normal mode [21], [39], [40], the iENM method has implicitly utilized all normal modes [58] to explore the conformational transition from ELIC to GLIC, which cannot be accurately described by one or a few normal modes [14]. Therefore, it offers the possibility of dissecting the sequential motions of residues underlying the coupling between ligand binding and channel opening.

The iENM modeling does not explicitly include any bound ligand, which can be justified in light of the recent finding that the conformational pathway of the gating transition of nAChR is essentially unchanged whether or not agonists occupy the ligand-binding sites [71]. On the other hand, the lack of atomic details and solvent modeling would prevent iENM from probing the full details of channel gating dynamics (such as the hydration/dehydration of the pore).

Besides iENM, several alternative computational techniques [54]–[56] may be used to model the pathway of the gating transition. We have tried one of them (MinActionPath) [56], which seems to predict a different order of structural events than iENM (see Table S1). A systematic comparison between iENM and alternative methods will be desirable in the future.

In this study, we assume that the ELIC (GLIC) crystal structure captures the closed-channel (open-channel) form of pLGICs, although further studies are needed to establish the physiological relevance of the ELIC and GLIC structures. Notably, the TMD of ELIC is significantly different from that of the functionally closed structure of nAChR determined by electron microscopy [7]. Surprisingly, the latter resembles the TMD of GLIC. Indeed, an MD simulation of the nAChR structure found the channel pore to shrink further, which suggests that it is not at a fully closed state [35]. It is possible that the closed-state ensemble of ELIC is comprised of multiple conformations as represented by the ELIC structure [12] and the nAChR structure [7].

It is encouraging that we have found remarkable agreements between the iENM modeling based on ELIC/GLIC structures and the Φ-value analysis of α subunit of nAChR mutants [3], although the complexity and richness of the Φ-value analysis results is not reproduced by the iENM. Together, they support a conserved structural mechanism for ligand-gated channel opening in pLGICs. Nevertheless, given the sequential and structural differences between ELIC/GLIC and nAChRs, one should be cautious when using ELIC/GLIC as modeling system to guide functional studies of nAChRs. In the future, we will test the modeling results by performing Φ-value analysis directly on GLIC.

The intermediate conformations predicted by iENM obey the five-fold symmetry which is present in both ELIC and GLIC structures. It is, however, conceivable that structural fluctuations away from the minimal-energy iENM pathway may lead to asymmetric conformations as observed in a recent MD simulation of the GLIC structure [37]. Additionally, as a hetero-pentamer, the motions of five subunits of nAChRs are unlikely to follow the five-fold symmetry. A detailed modeling of the asymmetric motions in nAChRs awaits the solution of open- and close-channel conformations of nAChRs.

Our modeling is based on two crystal structures with different sequences, so a structural alignment is used to model the open form of ELIC using the GLIC structure. Although the uncertainty in alignment does not seem to significantly affect the results of our modeling, it is highly desirable to perform modeling using both closed and open forms of the same protein in the future.

## Methods

### Elastic network model (ENM) and normal mode analysis (NMA)

In an ENM, a protein structure is represented as a network of C_α_ atoms whose minimal-energy conformation is given by a crystal structure. A harmonic potential accounts for the elastic interaction between two C_α_ atoms that lie within a cutoff distance *R_c_* (set to 10 Å following [55]). The potential energy function of ENM is [48]

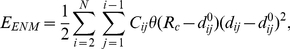
(1)where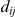 is the distance between the C_α_ atoms *i* and *j*, 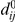 is the value of 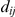
as given in a crystal structure, *N* is the number of C_α_ atoms, and 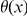 is the Heaviside function. 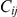
is the force constant of the spring between the C_α_ atoms *i* and *j*. 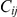
is set to 10 for chemically bonded residues [72] (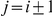), and 1 otherwise (the unit of 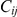
can be arbitrarily chosen without changing the modeling results).

The ENM potential energy can be expanded near a given conformation 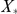 to the second order: 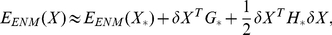
(2)where 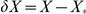, 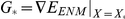
is the gradient of 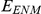 at 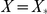, and
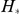
is the 3*N* 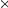
3*N* Hessian matrix given by
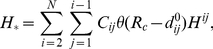
(3)where the elements of 3*N* 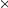
3*N* matrix
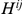
are given by 
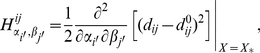
(4)where 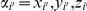 (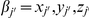) is the *x*, *y*, *z* component of the Cartesian coordinates of the C_α_ atom 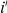
(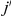). Note that the matrix elements of 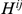
are nonzero only if (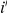 = 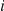
and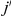 = 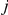), or (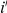 = 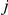
and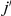 = 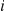), or (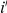 = 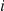
and 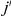 = 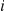), or (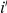 = 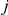
and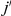 = 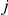).

From the hessian matrix *H*_1_ computed at 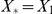 (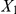 represents the C_α_ coordinates of the beginning conformation of a transition), we can solve 3*N* normal modes: the eigenvalue (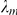) and eigenvector (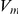) of mode *m* satisfy 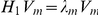. To evaluate the similarity between 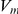 and the observed conformational change from 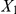
to
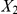
(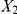 represents the C_α_ coordinates of the end conformation of a transition which is superimposed on 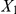), we compute the overlap coefficient 
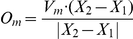
 for mode *m*, and the cumulative overlap 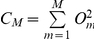
for the lowest *M* modes (after excluding the six translational/rotational modes). 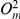
(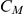) gives the percentage of the observed conformational change captured by mode *m* (the lowest *M* modes).

### Transition pathway modeled by iENM

We consider an ***arbitrary*** double-well potential function 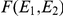 with two minima at the beginning and end conformations of a transition. It satisfies: 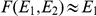 if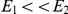, and 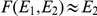 if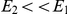, where 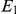 and 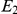 are two single-well potentials. Remarkably, the transition pathways generated by iENM (see below) are independent of the mathematic form of 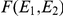 which varied in previous studies [54], [55]. The saddle points (SP) of 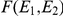 are solved as follows 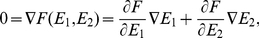
(5)which is equivalent to solving the following equation (after setting 
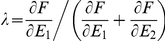
)
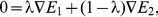
(6)where λ is a parameter of interpolation that varies from 1 to 0 (assuming 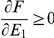 and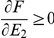). Therefore, the problem of solving SP for the double-well potential function 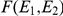 is converted to the problem of minimizing a linearly interpolated potential function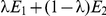. Alternatively, Eq. 6 gives a set of minimal-energy crossing points between 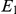 and 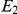 where 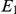 = 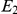
is at minimum.

Following the above general formulation, we have proposed an iENM protocol [58] based on a double-well potential function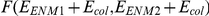, where 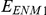 and 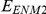
are two ENM potential functions (see Eq. 1) based at the beginning and end conformations of a transition, and 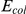 is a steric collision energy defined as follows:
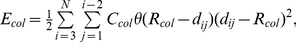
(7)where 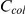 = 10, 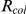 is the minimal distance between the C_α_ atoms of non-bonded residues in the beginning and end conformations of the transition (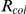
∼3 Å for the ELIC and GLIC structures). The chemically bonded residue pairs (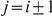) are excluded from the summation in Eq. 7. The addition of 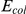
penalizes steric collisions between residues whose C_α_ atoms are within a distance of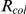. For the gating transition studied here, steric collisions are not serious so the addition of 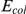 is not essential in determining the transition pathway.

With the addition of the collision energy, the SPs are solved by setting 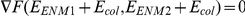 which is equivalent to solving the following SP equation (the SP is represented by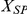):
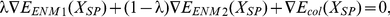
(8)As λ varies from 1 to 0, 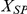 traces a pathway that connects the beginning and end conformations of a transition. Because this pathway passes all possible SPs, it gives a ‘universal’ minimum-energy path regardless of the mathematic form of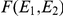 [55], [58]. iENM will output the above pathway as the predicted pathway for the given transition.

We solve Eq. 8 by finding the minima of the linearly interpolated potential function 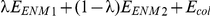 using the Newton-Raphson algorithm (for details, see [58]):

### Quantification of motional order of individual residues during a transition

Following [57], a fractional progress parameter 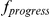
(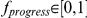) is defined for an intermediate conformation along a transition pathway: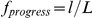, where *l* is the length of the part of the pathway from the beginning conformation to the intermediate conformation, while *L* is the total length of the pathway from the beginning conformation to the end conformation. The length of a pathway is computed approximately by summing up RMSDs between consecutive conformations along the pathway.

**Figure 4:**
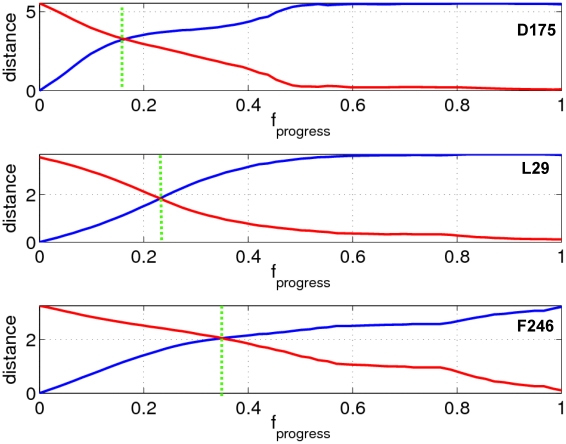
The distances (in Å) to the beginning/end C_α_ positions of residues D175 (in loop C), L29 (in loop 2) and F246 (at the physical gate of M2 helix, see [12]) in the ELIC/GLIC structure (shown as blue/red curves). These residues are shown as spheres in Fig. 1a. The 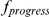
value at the crossing point of the two curves is used to assess the motional order of these residues (see Methods).

To quantify the motional order of individual residues along the iENM pathway, we use the following procedure [58]: first, we determine for each residue its ‘crossover conformation’ on the iENM pathway where the residue's C_α_ atom is at equal distance from its beginning and end positions of the transition (see Fig. 4); next, we assign to each residue the 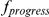
value of its crossover conformation. Residues with low (high)
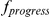
values, as colored by red (blue) in Fig. 1a, move early (late) during the transition.

### Structural alignment of ELIC and GLIC structures

We use the DALI server [73] to perform the structural alignment of the ELIC structure (PDB code: 2VL0) and the GLIC structure (PDB code: 3EHZ). 282/306 residues in each ELIC subunit are structurally aligned with Z score 22.0 (see Fig. S1 of Supporting Information). Only 24 residues in the ECD of ELIC cannot be aligned to GLIC ― including residues 59–62, 67–70, 132 (in loop B), 151–157 (in loop 9), 176–183 (in loop C). Most of them correspond to insertions in the ECD of ELIC compared with GLIC. For the lack of C_α_ coordinates for these unaligned residues in the open conformation, we do not include their non-bonded interactions in the ENM potential function (*E_ENM2_*) constructed from the open conformation.

To check the dependence of iENM modeling on structural alignments, we have tried two alternative structural alignment techniques (SSAP [74], CE [75]), which have obtained slightly different alignments in ECD than DALI. We have got very similar results in the motional order of residues by using these alternative structural alignments.

We have also applied iENM modeling to another GLIC crystal structure (PDB code: 3EAM) and obtained essentially same results.

We structurally align the entire pentamer (except the above mentioned unaligned residues) to account for the motions of all parts equally (both within and between ECD and TMD domains).

## Supporting Information

Figure S1 Result of structure-based sequence alignment between ELIC and GLIC by Dali. The key loops (loops A, B, C, 2, 7, 9) are highlighted in yellow, and the trans-membrane helices (M1–M4) are highlighted in cyan.
(0.03 MB DOC)

Table S1 Comparison of iENM with mixed-ENM and MinActionPath. To compare our method (iENM) with two alternative methods --- mixed-ENM [55] and MinActionPath [56], we have modeled the ELIC-to-GLIC transition using mixed-ENM and MinActionPath, and then analyzed their pathways using the *fprogress* parameter. We have found that they predicted different order of structural events than iENM (see Table S1), which does not compare well with the order deduced from experimental Φ values.
(0.03 MB DOC)

Video S1 In this movie, several key loops are colored differently (loop A: red, loop B: orange, loop C: yellow, loop 2: green, loop 7: pink, loop 9: cyan).
(1.39 MB MPG)
