# Supplementary material for: Decrypting the Sequence of Structural Events during the Gating Transition of Pentameric Ligand-Gated Ion Channels Based on an Interpolated Elastic Network Model
Source: PLoS Comput Biol. 2011 Jan 6;7(1):e1001046. doi: 10.1371/journal.pcbi.1001046 (PMC3017109; doi:10.1371/journal.pcbi.1001046)
Supplement: Figure S1 — Result of structure-based sequence alignment between ELIC and GLIC by Dali. The key loops (loops A, B, C, 2, 7, 9) are highlighted in yellow, and the trans-membrane helices (M1–M4) are highlighted in cyan. (0.03 MB DOC) [file pcbi.1001046.s001.doc]

DSSP lllllllLEEEEELEEEEEEEEEELLLLEEEEEEEEEEEEELHhHLLLLlllLLLLEEL-

GLIC pppiadePLTVNTGIYLIECYSLDDKAETFKVNAFLSLSWKDRrLAFDPvrsGVRVKTY- 59

ident | | | | | || | | |

ELIC -------PVDVSVSIFINKIYGVNTLEQTYKVDGYIVAQWTGK-PRKTP---GDKPLIVe 49

DSSP -------LEEEEEEEEEEEEEEEELLLLEEEEEEEEEEEELLL-LLLLL---LLLLEEEl

**Loop 2**

DSSP ---LLLL----LLLLLEELLLLLLLLEEEEEEEEELLLLEEEEEEEEEEEEELLLLHHHH

GLIC ---EPEA----IWIPEIRFVNVENARDADVVDISVSPDGTVQYLERFSARVLSPLDFRRY 112

ident | | | || | ||| | | || |||

ELIC ntqIERWinngLWVPALEFINVVGSPDTGNKRLMLFPDGRVIYNARFLGSFSNDMDFRLF 109

DSSP hhhHHHHhhllLLLLLEEELLEEEEEEELLLLEEELLLLLEEEEEEEEEEEELLLLLLLL

**Loop A Loop 7**

DSSP HHLEEEEEELLE-EELLllLEEEEELhhhLLLLLLL----lllLLEEEEEEEEEeeEEEE

GLIC PFDSQTLHIYLI-VRSVdtRNIVLAVdleKVGKNDD----vflTGWDIESFTAVvkPANF 167

ident ||| | | | |

ELIC PFDRQQFVLELEpFSYN--NQQLRFS---DIQVYTEnidneeiDEWWIRKASTH--ISDI 162

DSSP LLLEEEEEEEEEeLLLL--LEEEEEE---EEELLLLlllllllLLEELLLLLLL--EEEE

**Loop B Loop 9**

DSSP ELL--------LLEEEEEEEEEEEEELLLLHHHHLHHHHHHHHHHHHHHHHLLLHHHHHH

GLIC ALE--------DRLESKLDYQLRISRQYFSYIPNIILPMLFILFISWTAFWSTSYEANVT 219

ident | | | ||| | || || |

ELIC RYDhlssvqpnQNEFSRITVRIDAVRNPSYYLWSFILPLGLIIAASWSVFWLESFSERLQ 222

DSSP ELLllllllllLLEEEEEEEEEEEEELLHHHHHHLHHHHHHHHHHHHLLLLLLLHHHHHH

**Loop C M1**

DSSP HHHHHHHHHHHHHHHHHHLLLLLLLLLHHHHHHHHHHHHHHHHHHHHHHLLLLlLLLLHH

GLIC LVVSTLIAHIAFNILVETNLPKTPYMTYTGAIIFMIYLFYFVAVIEVTVQHYLkVESQPA 279

ident | || || | | | | | |

ELIC TSFTLMLTVVAYAFYTSNILPRLPYTTVIDQMIIAGYGSIFAAILLIIFAHHR-QANGVE 281

DSSP HHHHHHHHHHHHHHHHHHHLLLLLLLLHHHHHHHHHHHHHHHHHHHHHHHLLL-LLLLLL

**M2 M3**

DSSP HHHHHHHhHHHHHHHHHHHHHHHHHHhhlll

GLIC RAASITRaSRIAFPVVFLLANIILAFlffgf 310

ident | | | ||| || |

ELIC DDLLIQR-CRLAFPLGFLAIGCVLVI----- 306

DSSP LLLLLLL-LLLLLLLLLLLLLLLLLL-----

**M4**

**Fig** **S1**. Result of structure-based sequence alignment between ELIC and GLIC by Dali. The key loops (loops A, B, C, 2, 7, 9) are highlighted in yellow, and the trans-membrane helices (M1-M4) are highlighted in cyan.
