# Supplementary material for: Decrypting the Sequence of Structural Events during the Gating Transition of Pentameric Ligand-Gated Ion Channels Based on an Interpolated Elastic Network Model
Source: PLoS Comput Biol. 2011 Jan 6;7(1):e1001046. doi: 10.1371/journal.pcbi.1001046 (PMC3017109; doi:10.1371/journal.pcbi.1001046)
Supplement: Table S1 — Comparison of iENM with mixed-ENM and MinActionPath. To compare our method (iENM) with two alternative methods --- mixed-ENM [55] and MinActionPath [56], we have modeled the ELIC-to-GLIC transition using mixed-ENM and MinActionPath, and then analyzed their pathways using the fprogress parameter. We have found that they predicted different order of structural events than iENM (see Table S1), which does not compare well with the order deduced from experimental Φ values. (0.03 MB DOC) [file pcbi.1001046.s002.doc]

**Table S1** Comparison of iENM with mixed-ENM and MinActionPath:

| key motif | $\left\langle f_{progress}\right\rangle$ | | |
| --- | --- | --- | --- |
|  | iENM | mixed-ENM | Min-Action-Path |
| loop A | 0.18 | 0.36 | 0.34 |
| loop B | 0.36 | 0.53 | 0.49 |
| loop C | 0.20 | 0.31 | 0.48 |
| loop 2 | 0.22 | 0.39 | 0.39 |
| loop 7 | 0.28 | 0.34 | 0.53 |
| loop 9 | 0.27 | 0.38 | 0.45 |
| M2 | 0.30 | 0.50 | 0.52 |
| M2-M3 linker | 0.35 | 0.38 | 0.36 |
| M4 | 0.39 | 0.67 | 0.67 |
| M3 | 0.43 | 0.61 | 0.65 |
| Inferred order | loop C🡪loop 2🡪loop7 🡪M2🡪M2-M3 linker 🡪M4🡪M3 | loop C🡪loop 7🡪M2-M3 linker🡪loop 2🡪M2🡪M3 🡪M4 | M2-M3 linker🡪loop 2 🡪loop C🡪M2🡪loop 7🡪 M3🡪M4 |
